# Prevalence and Determinants of Geohelminthiasis among School-Age Children in Jimma City, Ethiopia

**DOI:** 10.1155/2023/8811795

**Published:** 2023-11-27

**Authors:** Ahmed Zeynudin, Teshome Degefa, Sultan Suleman, Abdulhakim Abamecha, Zuber Hajikelil, Andreas Wieser

**Affiliations:** ^1^School of Medical Laboratory Sciences, Institute of Health, Jimma University, Jimma, Ethiopia; ^2^School of Pharmacy, Institute of Health, Jimma University, Jimma, Ethiopia; ^3^Department of Medical Laboratory Sciences, Wolkite University, Wolkite, Ethiopia; ^4^Division of Infectious Diseases and Tropical Medicine, University Hospital, Ludwig-Maximilian-Universitat (LMU) Munich, Munich 80802, Germany

## Abstract

School-age children (SAC) are at a higher risk of geohelminth or soil-transmitted helminth (STH) infections due to their practice of walking and playing barefoot, lack of adequate sanitary facilities, and poor personal hygiene. In Ethiopia, periodic deworming has been implemented since 2013 with the aim of interrupting the transmission of STH in children by 2025. To evaluate the likely success of such a control program, it is crucial to monitor the transmission of STH, especially in peri-urban settings where environmental sanitation is modest. The aim of this study was to determine the prevalence and determinants of STH infections among SAC in peri-urban areas of Jimma City, Southwestern Ethiopia. A community-based cross-sectional study was conducted in five peri-urban *Kebeles* of Jimma City from July to September, 2021. Systematic random sampling was used to select 522 households with at least one child, and 478 children (5–15 years old) were recruited randomly from the households. Data on sociodemographic and potential risk factors were collected using a structured questionnaire. Stool samples from each study participant were collected and examined microscopically using the Kato–Katz technique. Multivariate logistic regression model was used to identify risk factors associated with STH infections. The prevalence of any STH among SAC was 23.4%, with *Ascaris lumbricoides* being the predominant STH species (15.7%), followed by *Trichuris trichiura* (9%) and hookworm (2.1%). Most (86.6%) of the STH-positive SAC had a single infection and a light infection intensity (88.2%), with a mean intensity of 367.4 eggs per gram. Location of *Kebele* (AOR = 2.73; 95% CI: 1.21–6.16, *p*=0.016), lack of hand washing after defecation (AOR = 6.39; 95% CI: 3.16–12.95, *p* < 0.001), untrimmed fingernails (AOR = 2.65; 95% CI: 1.56–4.51, *p* < 0.001), and lack of previous deworming (AOR = 2.90; 95% CI: 1.47–5.74, *p*=0.002) were significant predictors for STH infections among SAC. In conclusion, the study revealed that STH infections are significant health problem in the peri-urban areas of Jimma City. Strengthening periodic deworming and improving children's hygiene through health education are required to reduce the transmission.

## 1. Background

Soil-transmitted helminths (STHs) or geohelminths are the most widespread human parasitic infections in tropical and subtropical regions of the world where hygienic practices are poor and sanitary facilities are scarce [1–3]. Globally, more than 4.5 billion people are at risk of STH infection, with about 25% of the world population estimated to have been infected with at least one STH species leading to over 1.94 million disability-adjusted life years lost due to STH-related morbidity every year [4, 5]. Sub-Saharan Africa (SSA), Latin America, India, China, and East Asia are the most affected regions, sharing the highest burden of STH infections [6]. *Ascaris lumbricoides*, *Trichuris trichiura*, and hookworms (*Necator americanus* and *Ancylostoma duodenale*) are the main parasitic species causing geohelminthiasis in the world [5].

Soil-transmitted helminths are mainly transmitted by eggs that are passed in the feces of infected people. The adult worms live in the intestine, where they produce thousands of eggs each day, which may contaminate the environment, vegetables, and other food items that lack adequate sanitation [7–9]. The infections are mostly abundant among communities, particularly children, living in areas where there is no access to basic infrastructural amenities such as potable water supply, hygiene, and sanitary facilities [10–12]. Children are the most vulnerable group for STH infections due to their practice of walking and playing barefoot, frequent contact with contaminated soil, and poor personal hygiene [13].

High-intensity STH infections and polyparasitism may cause severe clinical manifestations in children, including abdominal pain, diarrhea, blood and protein loss, anemia, and rectal prolapse [5]. Chronic and subclinical infections could lead to stunting, reduced body mass index, and impaired physical and cognitive development in children [14]. Apart from the direct morbidity, STH infections could lead to low school enrollment, reduced school performance, and absenteeism [15], ultimately slowing educational advancement and economic development of endemic countries.

In SSA, helminth infections account for up to 85% of the neglected tropical diseases' burden, with up to one-third of the SSA population infected by one or more STH species [16]. Children, especially SAC, are the most affected in the region, with over 41 million children estimated to have been parasitized with at least one STH species [17]. In the past decades, there have been reports of a substantial reduction in the intensity of STH infections as a result of preventive chemotherapy and improved water, sanitation, and hygiene programs across Africa [17, 18]. Nevertheless, it remains a public health concern in many African countries and continues to cause major socioeconomic problems in the region [19].

Ethiopia is one of the SSA countries carrying the highest burden of STH infections, with an estimated 9 million preschool-aged children and 25 million SAC living in STH endemic areas [20, 21]. Since 2013, periodic mass drug administration (MDA) using single-dose albendazole (400 mg) or mebendazole (500 mg) has been widely implemented against STH in children in endemic areas of the country with the aim of eliminating STH-related morbidity by reducing moderate- and heavy-intensity infections to less than 1% by 2020 and interrupting the transmission by 2025 [20, 21]. Despite the national strategies implemented to lessen the burden of STH infections through preventive chemotherapy, the disease still remains a major public health concern in many parts of Ethiopia [22]. To evaluate the likely success of this control program, it is crucial to monitor the transmission of STH and identify the contributing factors, especially in peri-urban settings where environmental sanitation is modest. This study was conducted to determine the prevalence and determinants of STH infections among SAC in peri-urban areas of Jimma City, Southwestern Ethiopia.

## 2. Methods

### 2.1. Study Design and Setting

A community-based cross-sectional study was carried out in five peri-urban *Kebeles* (the smallest administration unit in Ethiopia) of Jimma City from July to September, 2021. Jimma City is located at 356 km southwest of Addis Ababa, the capital city of Ethiopia. The city is characterized by a semiarid type of climate with an average annual rainfall of 800–2,500 mm and a temperature range of 20–30°C [23]. The total population of Jimma City was estimated to be 205,384 in 2018 [24]. The city is administratively divided into 17 *Kebeles* (12 urban and 5 peri-urban *Kebeles*) [25]. The study area has been under biannual MDA since 2013.

### 2.2. Sample Size and Sampling Procedure

The sample size was determined using a single population proportion formula assuming STH prevalence of 21.7% obtained from the national mapping of STH and Schistosome infections in Ethiopia [22]. By using 95% confidence interval, a marginal error of 5%, and a design effect of 2, the total sample size was calculated to be 522. All of the five peri-urban *Kebeles* of Jimma City were included in the study. Systematic random sampling was used to select 522 households, and one school-aged child (5–15 years old) was selected for participation in the study from each household. A lottery method was used to select SAC from the households that had more than one child. The sample size was allocated proportionally to each study site based on the number of households in each *Kebele*.

### 2.3. Qualitative Data Collection and Processing

Data on sociodemographic characteristics of the SAC, personal hygiene practices, and other potential risk factors for STH infections were collected using a pretested structured questionnaire. The questionnaire was first prepared in English and then translated into the local language of the study area (Afaan Oromoo). After obtaining written informed consent from the household head of the SAC, trained community health workers conversant in the local language conducted the house-to-house survey to collect the qualitative data.

### 2.4. Stool Sample Collection and Examination

A single stool sample (about 5 g) was collected from each study participant using a clean and leak-proof screw cap container and labelled with a unique identification number. All collected stool samples were stored in a plastic bag and transported to the Medical Parasitology Laboratory of Jimma University within 2 hours. The samples were then processed using Kato–Katz smear and examined microscopically to detect ova of the STH and quantify the intensity of infections based on fecal egg count (eggs per gram) according to the World Health Organization (WHO) guidelines [26].

### 2.5. Data Analysis

Data were checked for completeness and entered into EpiData version 4.6 and then exported to SPSS version 25.0 for analysis. The dependent variable was the binary presence of any STH among the SAC. All independent variables including sociodemographic characteristics, personal hygiene practices, sanitation, and behavioral factors were treated as categorical variables and presented as frequencies and percentages. A chi-square test was used to identify any association between STH infections and the independent variables. Both bivariate and multivariable logistic regression analyses were used to identify the candidate variables and determinants of STH infections, respectively. Bivariate logistic regression analysis was used to identify variables with *p* values ≤0.25. Multivariable logistic regression analysis was performed to identify the predictors of STH infections among SAC. Adjusted odds ratio (AOR) with 95% confidence intervals was computed, and a *p* value less than 0.05 was considered statistically significant during the analysis.

## 3. Results

### 3.1. Sociodemographic Characteristics of the Study Participants

Out of 522 enrolled SAC, complete data (samples) were obtained from 478 (91.6%) children. Of the 478 SAC, 259 (54.2%) were males and 219 (45.8%) were females. The mean age of the study participants was 8.89 ± 2.83 years. The majority (88.1%) of the SAC were enrolled at school. Overall, 62% and 64.8% of the children's fathers and mothers had completed primary education, respectively. One-third of the fathers were engaged in agricultural activity, while 44.3% of mothers were housewives (Table 1).

### 3.2. Prevalence of STH among School-Age Children

**Figure 1:**
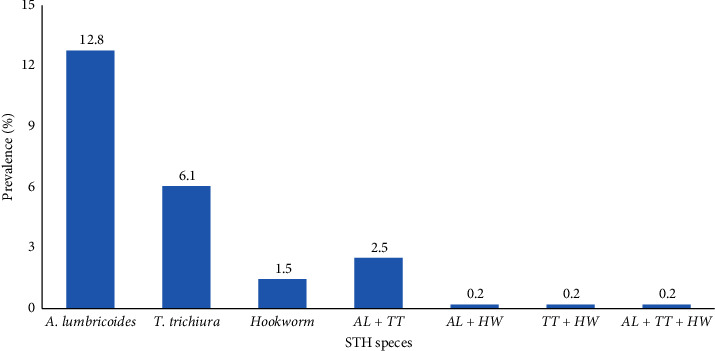
Prevalence of STH infections among school-age children in peri-urban *Kebeles* of Jimma City, Ethiopia. Note: AL, *A. lumbricoides*; TT, *T. trichiura*; HW, hookworm.

Overall, 140 (29.3%) and 112 (23.4%) of the SAC were positive for at least one species of intestinal helminths and STH, respectively. *Ascaris lumbricoides* was the most predominant STH species, detected in 15.7% of study participants, followed by *T. trichiura* (9.0%) and hookworm (2.1%). In addition to the STH, five cases of *Schistosoma mansoni*, thirty-one cases of *Hymenolepis nana*, six cases of *Enterobius vermicularis*, and one case of *Taenia* species were identified in the stool specimens. The majority (86.6%) of the SAC were infected by single species of STH, while double and triple infections were found in 12.5% and 0.9% of the SAC, respectively. Double infections were mainly due to *A. lumbricoides* and *T. trichiura* (Figure 1).

Most of the SAC infected with *A. lumbricoides* (82.7%) and *T. trichiura* (95.3%) and all infected with hookworm had a light infection intensity, and no heavy infection intensity was detected in any cases. The overall geometric mean fecal egg count for *A. lumbricoides*, *T. trichiura*, and hookworm was 688.8, 146.7, and 170.7 eggs per gram (EPG), respectively (Table 2)

### 3.3. Water Supply, Sanitation, and Hygiene- (WASH-) Related Conditions of SAC's Households

Overall, 97.1% of the SAC's households had access to an improved source of water for drinking. Protected well/spring was the most commonly used source of water by the households both for drinking and domestic use. Most (93.7%) of the households had private toilet facilities, while 4.8% of them reported that they shared toilet facilities with neighbors and 1.5% practiced open defecation. The majority (57.5%) of the households used improved toilet facilities (traditional pit latrines with slab, ventilated improved latrines, and flush latrines), but only 12.9% of them had hand washing facilities around the latrines. Most of the households practiced improper solid and liquid waste disposal systems (Table 3).

### 3.4. Factors Associated with STH Infections among SAC

Multivariable logistic regression analysis showed that location of Kebele, hand washing after defecation, status of the fingernails, and history of deworming had a significant association with STH infections in SAC (Table 4). Children living in Kofe *Kebele* were 2.7 (AOR = 2.73, 95% CI: 1.21–6.16) times more likely to be infected with STH compared to those residing in Ifabula *Kebele*. The prevalence of STH infections was 6.4 (AOR = 6.39, 95% CI: 3.16–12.95) times higher among children who often wash their hands after defecation as compared to those who always wash their hands after defecation. The odds of having STH were 2.6 (AOR = 2.65, 95% CI: 1.56–4.51) times higher among children who had untrimmed fingernails as compared to children who had trimmed their fingernails. Children who were not reportedly dewormed during the last MDA in 2020 were 2.9 (AOR = 2.90, 95% CI: 1.47–5.74) times more at risk of being infected with STH as compared to those who were treated (Table 4).

## 4. Discussion

In Ethiopia, intensive STH control through periodic MDA among children has been widely implemented with the aim of interrupting the transmission of STH in endemic areas by 2025. In this study, the overall prevalence of at least one species of STH among SAC in peri-urban areas of Jimma City was 23.4%, indicating that the transmission is still ongoing in the community at a level of public health concern. Recent studies conducted in Sekela, Western Ethiopia, and Blue Nile Basin, Northwest Ethiopia, have also reported a similar magnitude of STH infection prevalence among SAC [27, 28].

According to the WHO, STH endemic areas are classified into three transmission categories in line with the application of MDA in SAC [26]. These are high-risk areas where STH infection prevalence exceeds 50%, moderate-risk areas with parasite prevalence between 20% and 50%, and low-risk areas with STH prevalence below 20%. Based on this category, the current study areas would be classified into the moderate risk level, suggesting that control measures should be intensified to further reduce STH prevalence and transmission among the SAC.

The overall STH prevalence (23.4%) reported in this study is lower than the findings of earlier studies conducted in SAC in Jimma City (48.4% to 55%) [23, 29–31]. The lower STH prevalence documented in the current study might be due to improvements in sanitation practice. In the previous studies conducted in Jimma City, open field defecation was found to be one of the main factors contributing to STH transmission in SAC [30]. In this study, most (93.7%) of the SAC reported having latrines on their premises. Similarly, the overall STH prevalence documented in this study is lower compared to studies conducted in different localities of Ethiopia such as Chencha district (39.4%), Sigmo district (41.7%), Ejaji town (38.2%), Gamo Gofa zone (33.3%), and northwest parts of Ethiopia (32.3%) [32–36], as well as studies conducted in other countries such as Rwanda (77.7%), Nigeria (59.2%), Indonesia (33.8% to 57.2%), and Malaysia (78.1%) [37–41].

On the other hand, the overall prevalence of STH documented in this study is higher than that of studies conducted in Ambo town (12.6%), Goro (15.8%), and Babile town (0.47%) in Ethiopia [42–44], Zambia (14.4%) [45], and Kenya (17%) [46]. Such variations could be attributed to differences in personal hygiene, environmental sanitation, availability of adequate and safe water supply, and the socioeconomic status of the community [47]. Differences in diagnostic methods used for the detection of STH could also contribute to the variations in STH prevalence documented in different studies. The Kato–Katz technique was used in the present study, while McMaster and formol-ether concentration techniques were used in studies conducted in Ambo, Goro, and Babile [42–44]. In this study, the prevalence of STH varied even between the peri-urban *Kebeles*, indicating that STH prevalence could also vary between villages within a defined geographical area. Other studies have also documented a considerable and distinct spatial heterogeneity of STH infection prevalence and intensity [23, 48, 49].

In this study, *A. lumbricoides* was the most prevalent STH (15.7%) among the SAC, followed by *T. trichiura* (9%) and hookworm (2.1%). The predominance of *A. lumbricoides* is consistent with the findings of other studies conducted among SAC in different parts of Ethiopia [27, 29, 32, 34, 36, 42, 50] and abroad [37, 39, 45]. The higher prevalence of *A. lumbricoides* could be due to the fact that *Ascaris* eggs can withstand harsh environmental conditions more than the other species of STH and can easily sustain its transmission [51, 52]. In contrast to the present study, several other studies have reported the predominance of hookworm over the other STH species [33, 35, 43, 53, 54]. This difference might be due to differences in prevention measures used, climate variability, and other environmental factors. For instance, in settings where children often walk barefoot, hookworm is more prevalent as the infection occurs through skin penetration by larvae of the parasite [53]. In the current study, most of the SAC (94%) reported that they often wear shoes, and this might have contributed to the lower hookworm infection prevalence in this study. It has been documented that climate variability may also favor the occurrence of hookworm species due to its unique ability to undergo developmental arrest as dauer larvae in human tissues as a means of surviving extreme weather conditions [55–57]. Reports of the national STH prevalence survey also showed that *A. lumbricoides* is the predominant species in the highland and coldest regions of Ethiopia, such as Oromia and the southern region, while hookworm is more prevalent in the warmest regions, such as Benishangul-Gumuz and Gambella [22].

The reduction in moderate-to-heavy intensity infections is one of the indicators of the STH control program's success. STH is considered a public health problem by the WHO if at least 1% of SAC have moderate-to-heavy intensity infections [58]. In this study, about 88.3% of the STH-positive SAC had light-intensity infections, while the remaining 11.7% had moderate infections, indicating that STHs are still a public health problem in the study area. Although the overall prevalence of STH infections among SAC in this study is significantly reduced compared to the previous studies conducted in the study area before the commencement of MDA [29, 31], the prevalence of moderate-to-heavy STH infections in the current study is higher than the national report (2%) [22], suggesting that periodic deworming and other prevention measures should be strengthened in the area to further reduce the burden of STH.

In the present study, hand washing habit, status of the fingernail, deworming status, and location of the *Kebeles* were the key predictors of STH infections among SAC. Children who do not wash their hands after latrine use were six times more likely to be infected with STH compared to those who always wash their hands. This finding is consistent with studies conducted in different settings in Ethiopia [27, 28, 34, 59] and abroad [38, 41]. Moreover, children who had untrimmed fingernails were 2.6 times more likely to be infected with STH as compared to those who had trimmed their fingernails. Other studies have also reported a contribution of untrimmed fingernails to increased STH transmission among SAC [27, 35, 42]. This highlights the need to strengthen health education and awareness creation for SAC to improve their personal hygiene in order to interrupt the transmission of STH.

Children who had not taken anthelminthic drugs within one year of the study were three times more likely to be infected with STH as compared to their counterparts. A similar study was reported from India, where children who never took antihelminthics were more affected by STH than their counterparts [60].

The limitation of this study is that the prevalence and infection intensity of STH were determined by examination of a single stool specimen from each study participant. This may underestimate the actual STH infection prevalence among the SAC.

## 5. Conclusion

The findings of this study showed moderate level of STH infection prevalence among SAC living in the peri-urban areas of Jimma City. The higher prevalence of moderate-intensity STH infection (11.7%) above the target level (<1%) set by the national STH control program indicates that STHs are still a major health problem of the community of the peri-urban *Kebeles*. Lack of hand washing habit after latrine use, untrimmed fingernails, and lack of access to deworming were the key predictors of the STH infections among the SAC. Strengthening periodic deworming and provision of personal and environmental hygiene education and adequate sanitation facilities are required in order to eliminate STH as a public health problem and break transmission in children by 2025.

## Figures and Tables

**Table 1:** Sociodemographic characteristics of school-age children in peri-urban *Kebeles* of Jimma City, Ethiopia, July to September 2021.

Variables	Categories	Frequency	Percent (%)
Sex	Male	259	54.2
	Female	219	45.8

Age	5–10	351	73.4
	11–15	127	26.6

Educational status of SAC	Not enrolled to school	57	11.9
	Enrolled to school	421	88.1

Father's educational status	Cannot read and write	69	16.0
	Primary (1–8)	268	62.0
	Secondary (9–12)	67	15.5
	High school and above 12	28	6.5

Mother's education	Cannot read and write	94	19.8
	Primary (1–8)	308	64.8
	Secondary (9–12)	49	10.3
	High school and above 12	24	5.1

Father's occupation	Civil servant	123	28.5
	Merchant	69	16.0
	Daily laborer	97	22.5
	Farmer	134	31.0
	Others^*∗*^	9	2.1

Mother's occupation	Housewives	210	44.3
	Merchant	33	7.0
	Civil servant	64	13.5
	Daily laborer	78	16.5
	Farmer	89	18.8

^*∗*^Drivers and tailors.

**Table 2:** Intensity of STH infections among SAC in the peri-urban *Kebeles* of Jimma City, Ethiopia, July to September 2021 (*n* = 748).

STH species	Number of STH cases (%)	Infection intensity		
		Geometric mean EPG (95% CI)	Light (%)	Moderate (%)
*A. lumbricoides*	75 (15.7)	688.8 (457.1–1007.7)	62 (82.7)	13 (17.3)
*T. trichiura*	43 (9.0)	146.7 (107.4–202.0)	41 (95.3)	2 (4.7)
Hookworm	10 (2.1)	170.7 (107.0–282.2)	10 (100)	0
Overall	128	367.4 (278.0–480.0)	113 (88.3)	15 (11.7)

EPG: eggs per gram; CI: confidence interval.

**Table 3:** Water supply, sanitation, and hygiene conditions of SAC's households and distribution of STH among the SAC in the peri-urban *Kebeles* of Jimma City, Ethiopia.

Variables	Categories	STH prevalence		Total *n* (%)
		Positive *n* (%)	Negative *n* (%)	
*Hygiene*				
Hand washing before meal	Always	72 (17.6)	336 (82.4)	408 (85.3)
	Often	40 (57.1)	30 (42.9)	70 (14.6)
Hand washing after defecation	Always	70 (17.1)	340 (82.9)	410 (85.8)
	Often	41 (64.1)	23 (35.9)	64 (13.4)
	Sometimes	1 (25)	3 (75)	4 (0.8)
Washing/peeling fruits before eating	Always	41 (16.8)	203 (81.2)	244 (51.0)
	Often	47 (26.7)	129 (73.3)	176 (36.8)
	Sometimes	24 (41.4)	34 (58.6)	58 (12.1)
Status finger nail	Trimmed	48 (14.6)	280 (85.4)	328 (68.6)
	Untrimmed	64 (42.7)	86 (53.3)	150 (31.4)
Hand washing facility on premises	Available with soap and water	6 (22.2)	21 (77.8)	27 (6.0)
	Available without soap and water	9 (29.0)	22 (71.0)	31 (6.9)
	Not available	84 (21.5)	306 (78.5)	390 (87.1)
Hand washing after contact with soil	Always	54 (17.8)	250 (82.2)	304 (63.6)
	Often	47 (31.5)	102 (68.5)	149 (31.2)
	Sometimes	11 (47.8)	12 (52.2)	23 (4.8)
Fingernail biting/sucking habits	Yes	36 (31.9)	77 (68.1)	113 (23.6)
	No	76 (20.8)	289 (79.2)	365 (55.4)

*Sanitation*				
Latrine availability	Yes	99 (22.1)	349 (77.9)	448 (93.7)
	No	13 (56.7)	17 (43.3)	30 (6.3)
Status of the latrine facilities	Improved	41 (14.9)	234 (85.1)	275 (57.5)
	Unimproved	71 (35.0)	132 (65.0)	203 (42.5)
Shoe wearing habit	Always	30 (15.1)	169 (84.9)	199 (41.6)
	Often	70 (27.8)	182 (72.2)	252 (52.7)
	Sometimes	12 (46.2)	14 (53.8)	26 (5.4)

*Water source*				
Drinking water source	Protected/improved	111 (23.9)	353 (76.1)	464 (97.1)
	Unprotected	1 (7.1)	13 (92.9)	14 (2.9)
Water source for domestic use	Protected/improved	98 (23.2)	325 (76.8)	423 (88.5)
	Unprotected	14 (25.5)	41 (74.5)	55 (11.5)

*Waste disposal*				
Proper solid waste disposal	Yes	9 (16.7)	45 (83.3)	54 (11.3)
	No	103 (24.3)	321 (75.7)	424 (88.7)
Proper liquid waste disposal	Yes	3 (27.3)	8 (72.7)	11 (2.3)
	No	109 (23.3)	358 (76.7)	467 (97.7)

Improved toilet facilities include traditional pit latrines with slab, ventilated pit latrines, and flush latrines. Unimproved toilet facilities include pit latrines without slab. Protected water source includes tap water, tube hole/borehole, and protected well/spring. Unprotected water source includes unprotected hole/spring and surface water.

**Table 4:** Factors associated with soil-transmitted helminth infection among the SAC of peri-urban *Kebeles* of Jimma City, Ethiopia.

Variables	Categories	Soil-transmitted helminths		OR (95% CI)	*p* value	AOR (95% CI)	*p* value
		Positive *N*(%)	Negative *N*(%)				
Kebeles	Bore	40 (32.3)	84 (67.7)	1.67 (0.94–2.96)	0.082^*∗*^	1.43 (0.67–3.07)	0.355
	Kofe	20 (34.5)	38 (65.5)	1.84 (0.92–3.69)	0.085^*∗*^	2.73 (1.21–6.16)	0.016^*∗∗*^
	Hora Gibe	10 (25.6)	29 (74.4)	1.21 (0.52–2.80)	0.661	2.06 (0.79–5.34)	0.138
	Jiren	16 (11.4)	124 (88.6)	0.45 (0.23–0.89)	0.022^*∗∗*^	0.62 (0.28–1.37)	0.238
	Ifabula	26 (22.2)	91 (77.8)	1		1	

Age	5–10	84 (23.9)	267 (67.1)	1.11 (0.68–1.81)	0.668	—	—
	11–15	28 (22)	99 (78)	1			

Sex	Male	63 (24.3)	196 (75.7)	1.11 (0.73–1.71)	0.616	—	—
	Female	49 (22.4)	170 (77.6)	1			

Educational status SAC	Not enrolled at school	19 (33.3)	38 (66.7)	1.76 (0.97–3.20)	0.063^*∗*^	0.70 (0.30–1.60)	0.406
	Enrolled at school	93 (22.1)	328 (77.9)	1		1	

Decision-making autonomy in HH	Heads of the household	101 (26.7)	277 (73.3)	2.95 (1.51–5.74)	0.001^*∗*^	1.88 (0.88–4.04)	0.102
	A consensus of all family	11 (11)	89 (89)	1		1	

Mother's educational status	Cannot read and write	32 (34)	62 (66)	3.61 (1.00–13.00)	0.05^*∗*^	2.11 (0.50–8.91)	0.306
	Primary (1–8)	68 (22.1)	240 (77.9)	1.98 (0.57–6.84)	0.279^*∗*^	1.07 (0.27–4.20)	0.923
	Secondary (9–12)	9 (18.4)	40 (81.6)	1.57 (0.38–6.44)	0.528	0.89 (0.18–4.41)	0.888
	High school and above 12	3 (12.5)	21 (87.5)	1		1	

Mother's occupation	Housewife	39 (18.6)	171 (81.4)	1	1	1	
	Merchant	5 (15.2)	28 (84.8)	0.78 (0.28–2.15)	0.636	0.80 (0.21–2.92)	0.734
	Civil servant	12 (18.8)	52 (81.2)	1.01 (0.49–2.07)	0.974	1.21 (0.50–2.91)	0.668
	Daily laborer	38 (35.8)	68 (64.2)	2.59 (1.45–4.61)	0.001^*∗*^	1.71 (0.84–3.43)	0.133
	Farmer	18 (29.5)	43 (77.8)	1.91 (1.08–3.37)	0.026^*∗*^	1.04 (0.43–2.45)	0.934

Wealth status	Low	48 (31.8)	103 (68.2)	2.16 (1.27–3.69)	0.005^*∗*^	1.09 (0.50–2.39	0.816
	Medium	36 (21.3)	133 (78.7)	1.26 (0.72–2.18)	0.415	1.46 (0.65–3.25)	0.352
	High	28 (17.7)	130 (82.3)	1		1	

Family size	≤5	62 (26.8)	169 (73.2)	1		1	
	>5	50 (20.2)	197 (79.8)	0.69 (0.45–1.06)	0.09^*∗*^	0.82 (0.47–1.42)	0.481

Latrine availability	Yes	99 (22.1)	349 (77.9)	1		1	
	No	13 (56.7)	17 (43.3)	2.69 (1.26–5.74)	0.01^*∗*^	1.14 (0.34–3.82)	0.832

Hand washing after defecation	Always	70 (17.1)	340 (82.9)	1		1	
	Often	41 (64.1)	23 (35.9)	8.66 (4.89–15.34)	<0.001^*∗*^	6.39 (3.16–12.95)	<0.001^*∗∗*^
	Sometimes	1 (25)	3 (75)	1.62 (0.16–15.79)	0.678	2.11 (0.20–21.74)	0.531

Hand washing before meals	Always	72 (17.6)	336 (82.4)	1		1	
	Often	40 (57.1)	30 (42.9)	6.22 (3.63–10.56)	<0.001^*∗∗*^	1.65 (0.69–3.95)	0.260

Washing/peeling fruits before eating	Always	41 (16.8)	203 (81.2)	1		1	
	Often	47 (26.7)	129 (73.3)	1.80 (1.12–2.89)	0.015^*∗*^	0.72 (0.36–1.42)	0.349
	Sometimes	24 (41.4)	34 (58.6)	3.49 (1.87–6.50)	<0.001^*∗*^	1.009 (0.43–2.36)	0.983

Hand washing after contact with soil	Always	54 (17.8)	250 (82.2)	1		1	
	Often	47 (31.5)	102 (68.5)	2.13 (1.35–3.35)	0.001^*∗*^	0.98 (0.47–2.02)	0.956
	Sometimes	11 (47.8)	12 (52.2)	4.24 (1.77–10.12)	0.001^*∗*^	1.14 (0.314.22)	0.845

Shoe wearing	Always	30 (15.1)	169 (84.9)	1		1	
	Often	70 (27.8)	182 (72.2)	2.17 (1.34–3.48)	0.001^*∗*^	1.21 (0.67–2.21)	0.517
	Sometimes	12 (46.2	14 (53.8)	4.83 (2.03–11.44)	<0.001^*∗*^	2.82 (0.99–8.02)	0.051

Fingernail biting/sucking habits	Yes	36 (31.9)	77 (68.1)	1.78 (1.11–2.84)	0.016^*∗*^	1.08 (0.57–2.0)	0.809
	No	76 (20.8)	289 (79.2)	1		1	

Status of fingernail	Trimmed	48 (14.6)	280 (85.4)	1		1	
	Untrimmed	64 (42.7)	86 (53.3)	4.34 (2.78–6.78)	<0.001^*∗*^	2.65 (1.56–4.51)	<0.001^*∗∗*^

Presence of children <5 years of age in the household	Yes	60 (25.9)	172 (74.1)	1.30 (0.85–1.98)	0.224^*∗*^	1.26 (0.73–2.18)	0.405
	No	52 (21.1)	194 (78.9)	1			

Presence of domestic animal	Yes	25 (19.7)	102 (80.3)	0.744 (0.45–1.23)	0.246^*∗*^	1.09 (0.57–2.07)	0.793
	No	87 (24.8)	264 (75.2)	1		1	

Previous history of STH infection	Yes	59 (28.6)	147 (71.4)	1.65 (1.08–2.54)	0.02^*∗*^	1.52 (0.918–2.54)	0.103
	No	53 (19.5)	219 (81.5)	1		1	

Previous history of STH infection among families	Yes	71 (25.5)	207 (74.5)	1.50 (0.94–2.39)	0.083^*∗*^	0.60 (0.28–1.26)	0.182
	No	33 (18.5)	145 (81.5)	1		1	

History of deworming	Yes	74 (18.4)	329 (80.6)	1		1	
	No	38 (50.7)	37 (49.3)	4.56 (2.72–7.66)	<0.001^*∗*^	2.90 (1.47–5.74)	0.002^*∗∗*^

Key; ^*∗*^ = variables at *p* ≤ 0.25 in bivariate logistic regression, ^*∗∗*^predictor variables in multivariate logistic regression at *p* < 0.05 and 1 refers to reference group.

## Data Availability

The data supporting the conclusion of this article are included within the article. The raw data are available from the corresponding author upon reasonable request.

## Abbreviations

AOR: Adjusted odds ratio
CI: Confidence interval
EPG: Eggs per gram
MDA: Mass drug administration
OR: Odds ratio
SAC: School-age children
SSA: Sub-Saharan Africa
STH: Soil-transmitted helminth
WHO: World Health Organization.
